# Structural updates of alignment of protein domains and consequences on evolutionary models of domain superfamilies

**DOI:** 10.1186/1756-0381-6-20

**Published:** 2013-11-15

**Authors:** Eshita Mutt, Sudha Sane Rani, Ramanathan Sowdhamini

**Affiliations:** 1 National Centre for Biological Sciences (TIFR), UAS-GKVK Campus, Bellary Road, 560 065 Bangalore, India; 2 International Institute of Information Technology-Hyderabad, Gachibowli, 500032 Hyderabad, India; 3 Department of informatics, University of Oslo, Blindern, P.O box 10800316 Oslo, Norway

**Keywords:** PASS2 database, Protein domain, Length variations, Insertions, Deletions

## Abstract

**Background:**

Influx of newly determined crystal structures into primary structural databases is increasing at a rapid pace. This leads to updation of primary and their dependent secondary databases which makes large scale analysis of structures even more challenging. Hence, it becomes essential to compare and appreciate replacement of data and inclusion of new data that is critical between two updates. PASS2 is a database that retains structure-based sequence alignments of protein domain superfamilies and relies on SCOP database for its hierarchy and definition of superfamily members. Since, accurate alignments of distantly related proteins are useful evolutionary models for depicting variations within protein superfamilies, this study aims to trace the changes in data in between PASS2 updates.

**Results:**

In this study, differences in superfamily compositions, family constituents and length variations between different versions of PASS2 have been tracked. Studying length variations in protein domains, which have been introduced by indels (insertions/deletions), are important because theses indels act as evolutionary signatures in introducing variations in substrate specificity, domain interactions and sometimes even regulating protein stability. With this objective of classifying the nature and source of variations in the superfamilies during transitions (between the different versions of PASS2), increasing length-rigidity of the superfamilies in the recent version is observed. In order to study such length-variant superfamilies in detail, an improved classification approach is also presented, which divides the superfamilies into distinct groups based on their extent of length variation.

**Conclusions:**

An objective study in terms of transition between the database updates, detailed investigation of the new/old members and examination of their structural alignments is non-trivial and will help researchers in designing experiments on specific superfamilies, in various modelling studies, in linking representative superfamily members to rapidly expanding sequence space and in evaluating the effects of length variations of new members in drug target proteins. The improved objective classification scheme developed here would be useful in future for automatic analysis of length variation in cases of updates of databases or even within different secondary databases.

## Background

Protein domains are one of the fundamental building blocks of protein structures and have been used as a unit for structural classification of proteins. Protein domains in primary structural databases such as PDB (Protein Data Bank) [1] have been grouped according to structural hierarchy such as protein folds, superfamilies and families in databases like CATH (Class, Architecture, Topology, Homologous superfamily) [2] and SCOP (Structural Classification of Proteins) [3]. There are also secondary databases like PASS2 (Protein Alignments organised as Structural Superfamilies) [4-6] which follows the SCOP hierarchy and provide highly accurate structure based sequence alignments for protein domain superfamilies. It is widely accepted that protein domains which cluster under a superfamily generally adopt similar tertiary structure, in spite of having low sequence identity. Early analysis of SCOP database and the statistics of different versions of PASS2 database revealed the presence of overwhelming majority of single-membered superfamilies, thus clearly suggesting that the incoming protein structural entries could greatly alter the composition and size of previously accumulated superfamilies [3]. Aside from this, there has been no rigorous analysis of the influence of the incoming entries into primary databases, such as protein structural entries on the composition of dependent secondary databases. We have examined the effects of such transitions using “length variation” as a parameter.

The ability of some protein folds to tolerate large changes in sequence and length has been noted earlier and such length changes have been caused during evolutionary drifts [2]. This length changes have been caused by “indels” (insertions/deletions) in protein sequences which has in turn been used to follow updates of secondary databases derived from SCOP. Earlier studies by our group had examined the length variations in 353 multi-membered superfamilies from PASS2.2 database [4], using an objective algorithm called CUSP (Conserved Units of Structures in Proteins) [7], and analysed length variations and its consequences on functionality of protein domains [8]. Such analyses have been helpful to recognise and classify superfamilies into 64 “Length-deviant” (ones which can tolerate large {i.e. more than 30%} variations in domain length from the average domain length of that superfamily) and 24 “Length-rigid” (ones which are less tolerant i.e. <10% variations in domain length from the average domain length of that superfamily i.e. have similar sized members) superfamilies. Such length variations, caused by indels, were shown to play a principle role in introducing important evolutionary signatures in form of changing substrate specificity, altering domain interactions and sometimes even regulating protein stability [8]. This study is also important from the drug discovery perspective, whose pace can be enhanced by *apriori* knowledge about the effects and location of length variations in relation to the active sites or changes in passage of substrates (small drug-like molecules).

The backbone of CUSP analysis has been the database of structurally aligned protein domain superfamilies organised as PASS2.2 (version 2004), which was created to be in direct correspondence with SCOP 1.63. Many structure based alignment softwares were employed to create reliable alignments between distantly related proteins, as no two sequences in a superfamily of PASS2 had sequence identity of more than 40%. The updated version of PASS2.2 is now available as PASS2.3 (PASS2-2008) [6] which is in correspondence with SCOP 1.73. It has been anticipated that the presence of newer members will strongly influence the composition of previously recognised superfamilies, apart from setting new superfamilies to be realised. Furthermore, new and improved structure-based sequence alignments were employed in PASS2.3 (PASS2-2008) in comparison with PASS2.2 (PASS2-2004) version.

In comparison to PASS2.2 version, PASS2.3 not only went through improved methods of superposition and alignment, but the number of members and the classification of members into superfamilies also have gone through vital changes. While 377 superfamilies remained similar, many new superfamilies were formed or included in the recent version of PASS2 database (PASS2.3). In this study, the nature and source of variations in the number and composition of superfamilies, as they transition from PASS2.2 to PASS2.3 version, have been studied. Quality of structural alignments of protein domain structures have been compared between the two updates, using structure-based assessment parameters. Further, we have chosen 20 superfamilies, i.e. 10 length-rigid and 10 length-deviant superfamilies of PASS2.2 version, and the extent of their length variation has been compared as they appear in PASS2.3 version. We observe that whereas the quality of alignment has improved during the update, tracing changes between updates of protein domain superfamilies (from a length variation perspective or by other metrics) is non-trivial. We also recommend that, prior to large scale analysis on these vast databases, it may be worthwhile to screen them for breakup of superfamilies, inclusion of new members and their effects on the content of protein domain superfamilies.

## Methods

### Dataset collection

All 396 multi-membered superfamilies [2903 proteins] from PASS2.2 database [each superfamily having > =3 members and <40% identity within them] and 635 multi-membered superfamilies [5697 proteins] from PASS2.3 were used for this analysis. Older PASS2 (PASS2.2 - 2004 version) was in accordance with SCOP 1.63 version, while the newer PASS2 (PASS2.3 - 2008 version) was in accordance with SCOP 1.73. Initial exploration in the number and average domain sizes was performed between the versions. Previous analysis [7,8] on PASS2.2 version showed that superfamilies could be divided into “Length-deviant” and “Length-rigid” groups and ranked according to the extent of length variation with respect to average domain length. But in order to remove subjectivity involved in previous method, re-classification of above superfamilies was performed. They were then further subjected to length variation analysis.

### Length variation studies

Extent of length variation was calculated as explained in our earlier studies [7], where mean domain size for each superfamily was calculated by finding average lengths of each of the members; thereafter, deviation of the length of each member from the mean domain size was found and then after divided by the latter. The result was reported in percentage (%) thereafter.

Extent of length variation: ((domain length – mean length)/ mean length) *100

### Detailed analysis based on actual members

Update of PASS2 from PASS2.2 (2004) version to PASS2.3 (2008) version led to certain changes at coarse-grained level (superfamilies), as well as fine-grained (member) level. In order to understand the changes, all the 64 length-deviant superfamilies were used for analysis. Further, alignments of ten length-deviant superfamilies (Additional file 1: Table S1) and ten length-rigid superfamilies (Additional file 1: Table S2) were employed for comparison studies between the two versions of PASS2 database. Differences in number of members in each superfamily and average domain size between the PASS2 versions were also calculated. Literature was consulted to track the variations and its effects between the two versions for these ten length-deviant and length-rigid superfamilies. Statistical tests of significance between the two datasets were carried out using R package.

### Case study of length normal superfamilies which changed into length-rigid superfamilies

Out of 416 length-rigid superfamilies found by the improved classification (see Discussion section Improved scheme of classification for PASS2 superfamilies), length-deviant and length-rigid superfamilies from earlier analysis [7] were removed which left 173 superfamilies. These were checked manually and the superfamilies which retained 75% of members from the earlier PASS2 version were then taken up for further analysis. In this dataset, 16 superfamilies were from α-class, 11 superfamilies from β-class, 12 superfamilies from α/β class and rest 23 from α + β structural class (in accordance to SCOP structural class). Alignment files and PDB coordinate files were extracted from PASS2.2 and PASS2.3 (2004 and 2008 versions) datasets for the above superfamilies. Initial equivalences were obtained by using Joy4.0 package [9] while SSTeq (Secondary structural equivalences) and RMSD (Root Mean Square Deviation) was determined by MNYFIT (JOY3.2) package [10]. Results obtained were also shown to be an indicator of the alignment quality in the newer version of PASS2.

## Results

### Mapping of superfamilies between two versions of PASS2

**Figure 1:**
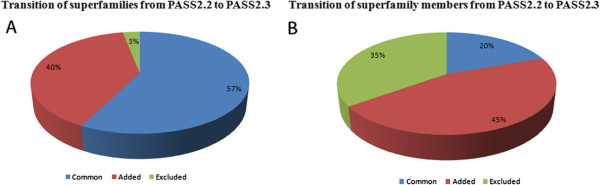
Tracking of transitions which have occurred between PASS2.2 and PASS2.3 with A) superfamilies and their B) members.

PASS2 versions were compared at superfamily level and also at individual member level [Additional file 2], where it was found that most of the superfamilies were present in both the versions but had differences in their member composition [Figure 1A, B]. The newly added members were found to have different domain lengths which changed the extent of length variation of that superfamily. It was also found that most of the superfamilies were sparsely populated (less than 10 members), unlike the densely populated ones like PDZ domain superfamily (with 50 members). Further on, the superfamilies were classified in distinct groups based on the extent of their length variations and the classification scheme was formulated so as not to bias the sparsely populated superfamilies.

**Figure 2:**
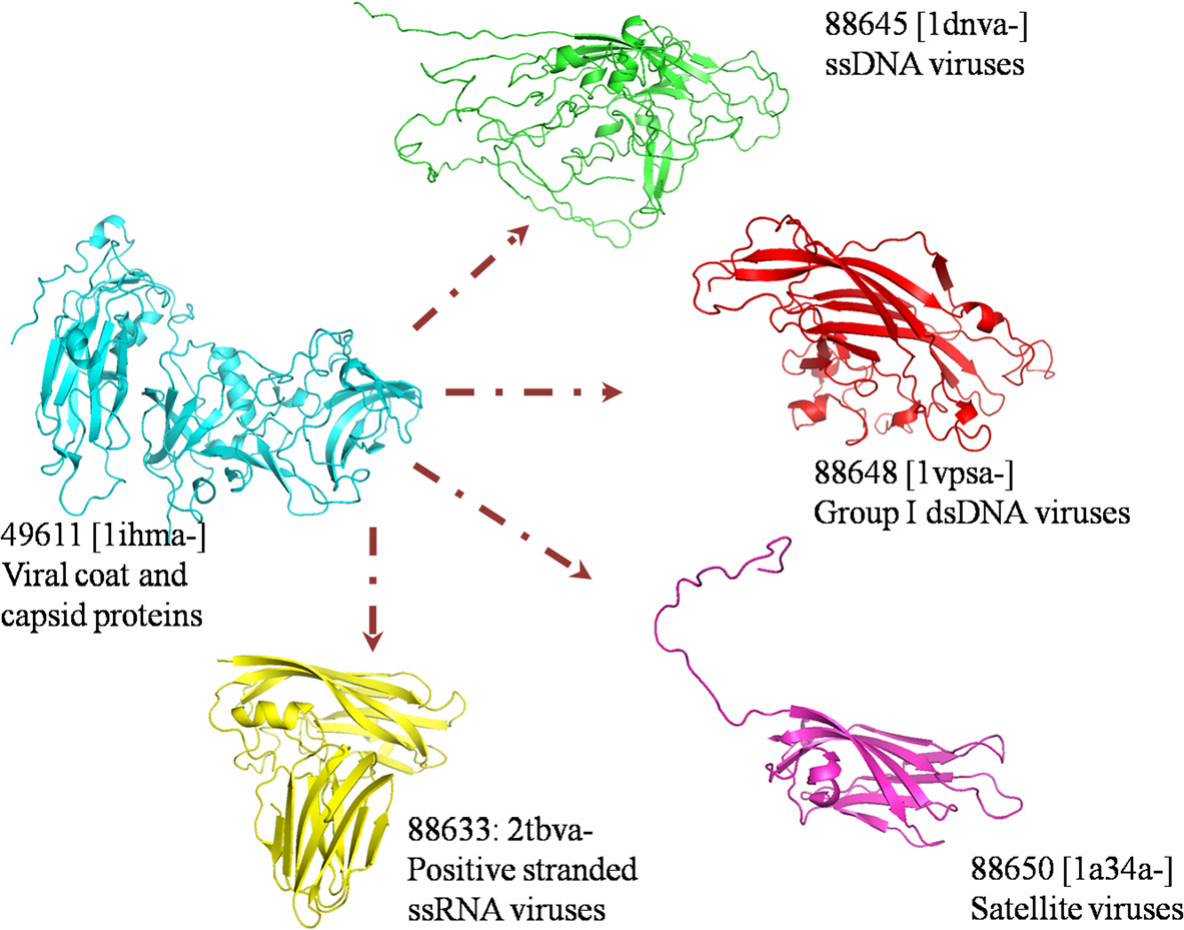
Illustration of splitting of viral protein superfamily [SCOP code: 49611] into four different superfamilies in PASS2_v2008.

At a coarser superfamily-based level, analysis showed that 20 superfamilies which were present in our earlier analysis [7] of 396 superfamilies were absent from the recent PASS2.3 set while 259 new superfamilies had been introduced. Overall, 377 superfamilies were common between the two datasets. [Figure 1A] While the addition of superfamilies may have been caused by inclusion of newer PDB structures at SCOP level or breakup of old superfamilies, exclusion may have been due to stringent cut-off at the PASS2 entry level. A case study may be the ETFP adenine nucleotide binding superfamily (SCOP code: 52431) which had been merged with Adenine nucleotide α-hydrolase-like superfamily (SCOP code: 52402); while another interesting example can be of the two superfamilies namely Viral coat and capsid proteins (SCOP code: 49611) and Ovomucoid/PC-1 like inhibitors (SCOP code: 57467) which had split into smaller superfamilies. While the former (SCOP code: 49611) had split into four superfamilies (SCOP code: 88633, 88645, 88648 and 88650) [Figure 2], the latter (SCOP code: 57467) had split into two superfamilies (SCOP code: 100895 and 100897). Apart from above three superfamilies, others had dropped out many members, which may have been due to the <40% sequence identity cut-off introduced in the PASS2 methodology; hence they became two-membered superfamilies (which have not been covered presently in this study).

### Statistics at superfamily level

**Figure 3:**
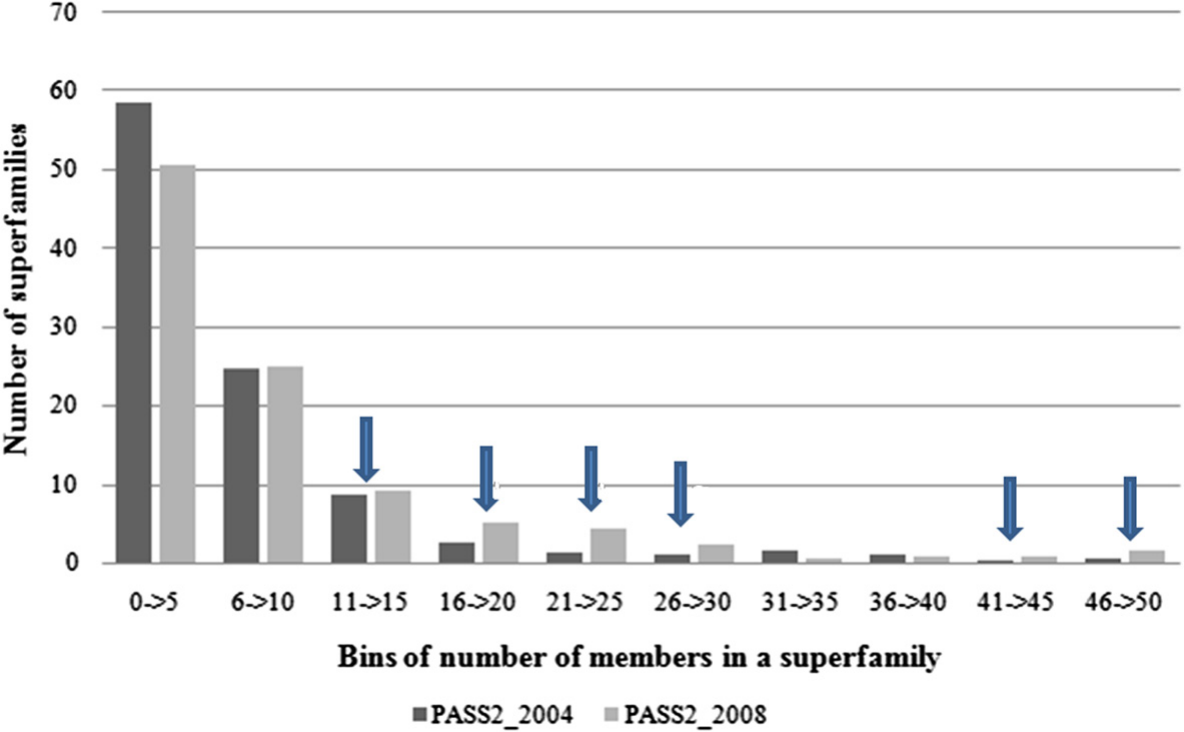
Distribution of number of superfamilies with respect to frequency of members in each superfamily (categorised as different bins).

Statistical inferences were drawn from all 396 multi-membered superfamilies [2903 domains] from PASS2.2 database [each having > =3 members and <40% identity within them] and 635 superfamilies [5697 domains] from PASS2.3 which have been used for this analysis. The simplistic approach to find differences between two datasets would have been to evaluate the number of members in superfamilies, which was determined and illustrated [Figure 3]. It shows that less-populated superfamilies (with less than 5 members) have decreased in PASS2.3 version, while highly populated superfamilies (in terms of 46 to 50 members) have increased [marked by arrows in Figure 3]. Though the increase is marginal, this can have an effect on classification of superfamilies based on their extent of length variation.

**Figure 4:**
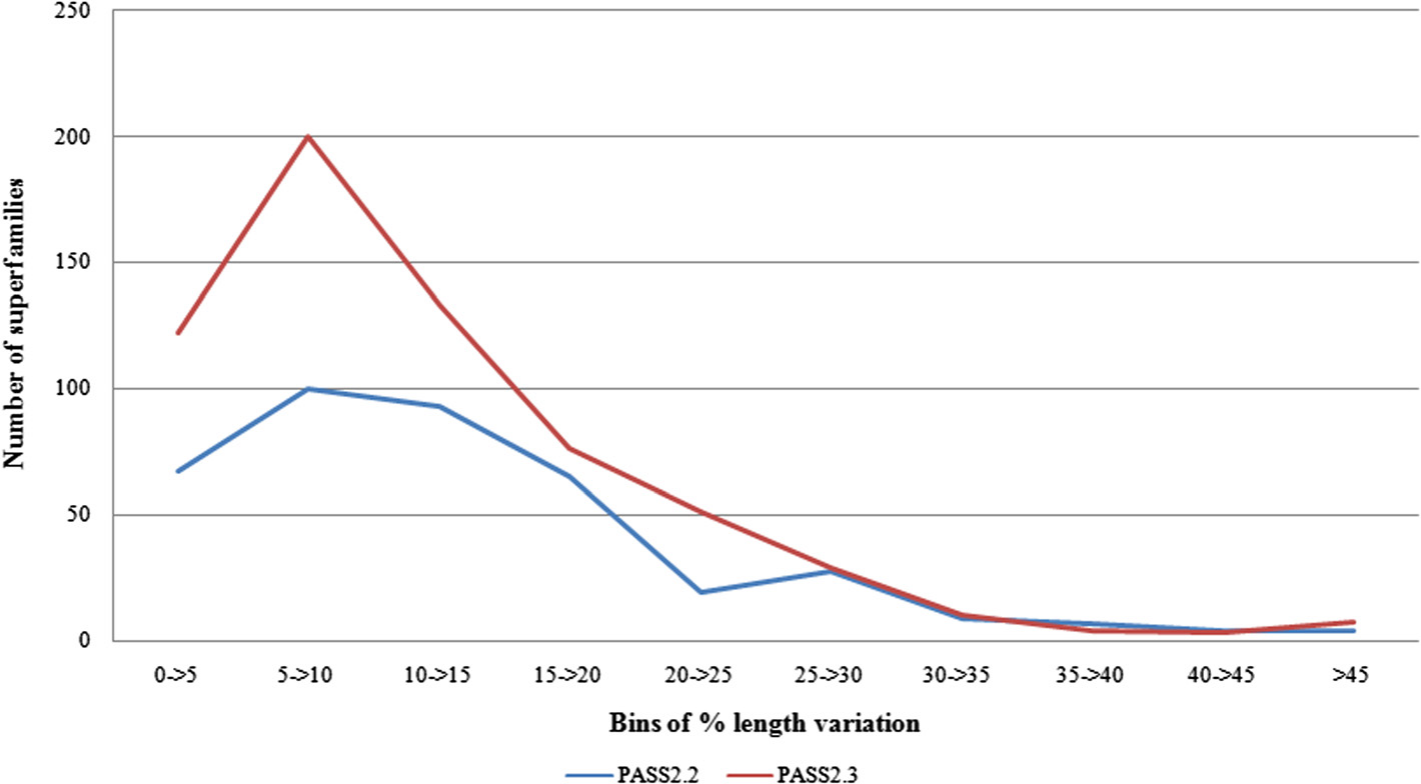
Distribution of % length variations into bins for PASS2.2 (2004) [in blue] and PASS2.3 (2008) [in red] (with respect to number of superfamily and number of members).

Next, the length variation patterns between the two datasets (with respect to the average domain size of a superfamily) were compared across each member and each superfamily for both database updates [Figure 4]. Comparison of the distribution of length variations between PASS2.2 and PASS2.3 versions showed that this graph had a single peak at 5- > 10% bin in PASS2.2 version and otherwise had a smooth slope, whereas two peaks (at 5- > 10% bin and 25- > 30% bin) were observed for PASS2.3 version. Further, the length-rigid character (presence of similar sized members) was enhanced in the 5- > 10% bin, and length-deviant character (presence of members with varying lengths) was found for a small group of nine superfamilies only. This clearly suggested that, in the PASS2.3 dataset having length-rigid and length-deviant superfamilies, superfamilies have become more rigid in nature and i.e. similar-sized members in terms of domain length have increased.

**Figure 5:**
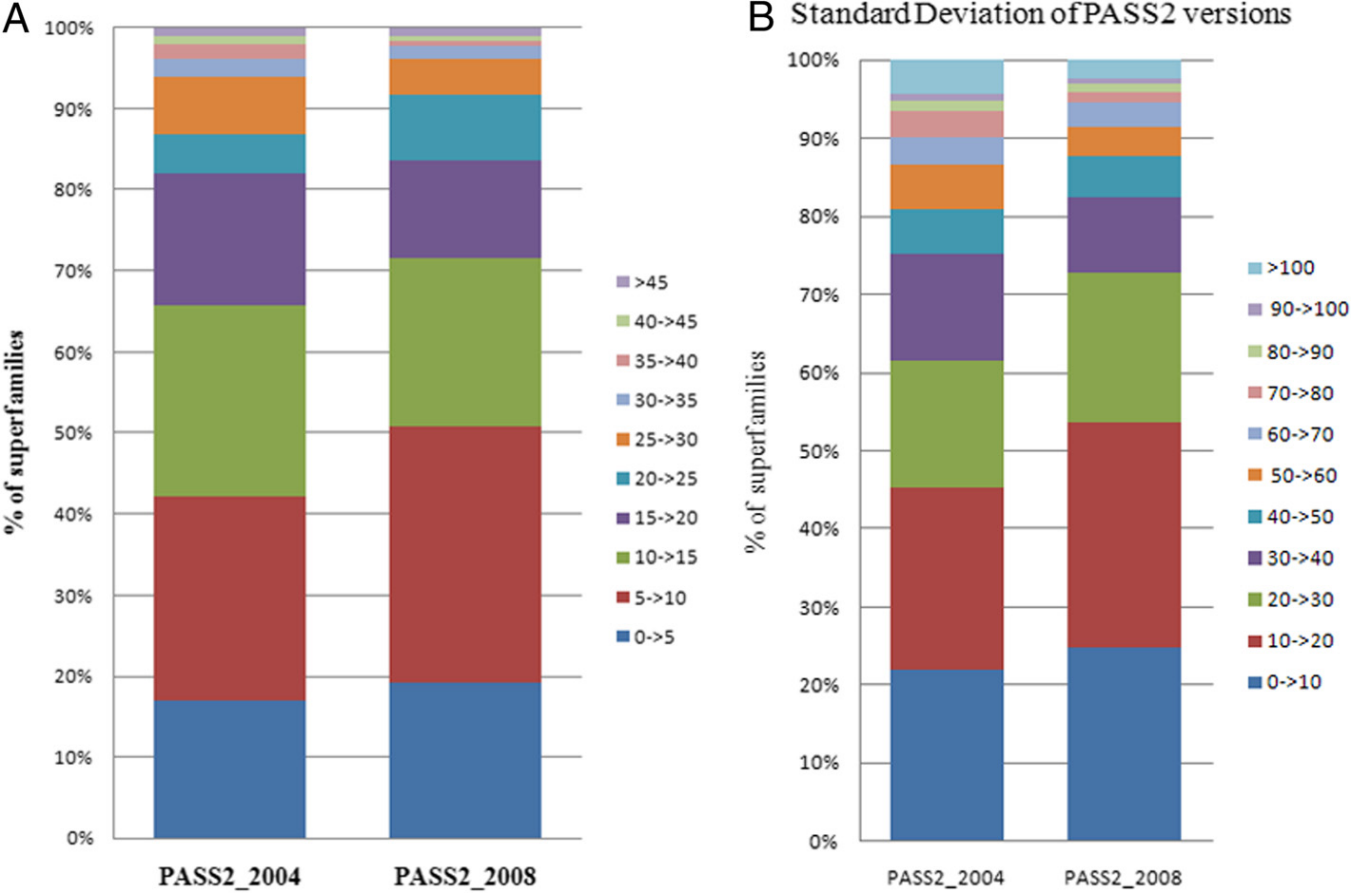
Distribution of (A) length variations (Units in %) and (B) standard deviations for PASS2.2 and PASS2.3 versions (3 or more member superfamilies considered).

Further, the length distribution was presented in bar-graph format [Figure 5A] and standard deviation of the length variation was also performed and depicted as bar-graph [Figure 5B]. In this bar-graph representation with% of superfamilies [Figure 5A], it could be clearly seen that the number of superfamilies within 0-5% and 10- > 15% length variation bins have grown from 2004 to 2008 datasets, while 25- > 30% and other higher bins have decreased. The same points were confirmed by the standard deviation study performed on the two datasets. Increase in rigidity (tendency to have similar length domain members) and decrease in deviant nature was again highlighted.

**Figure 6:**
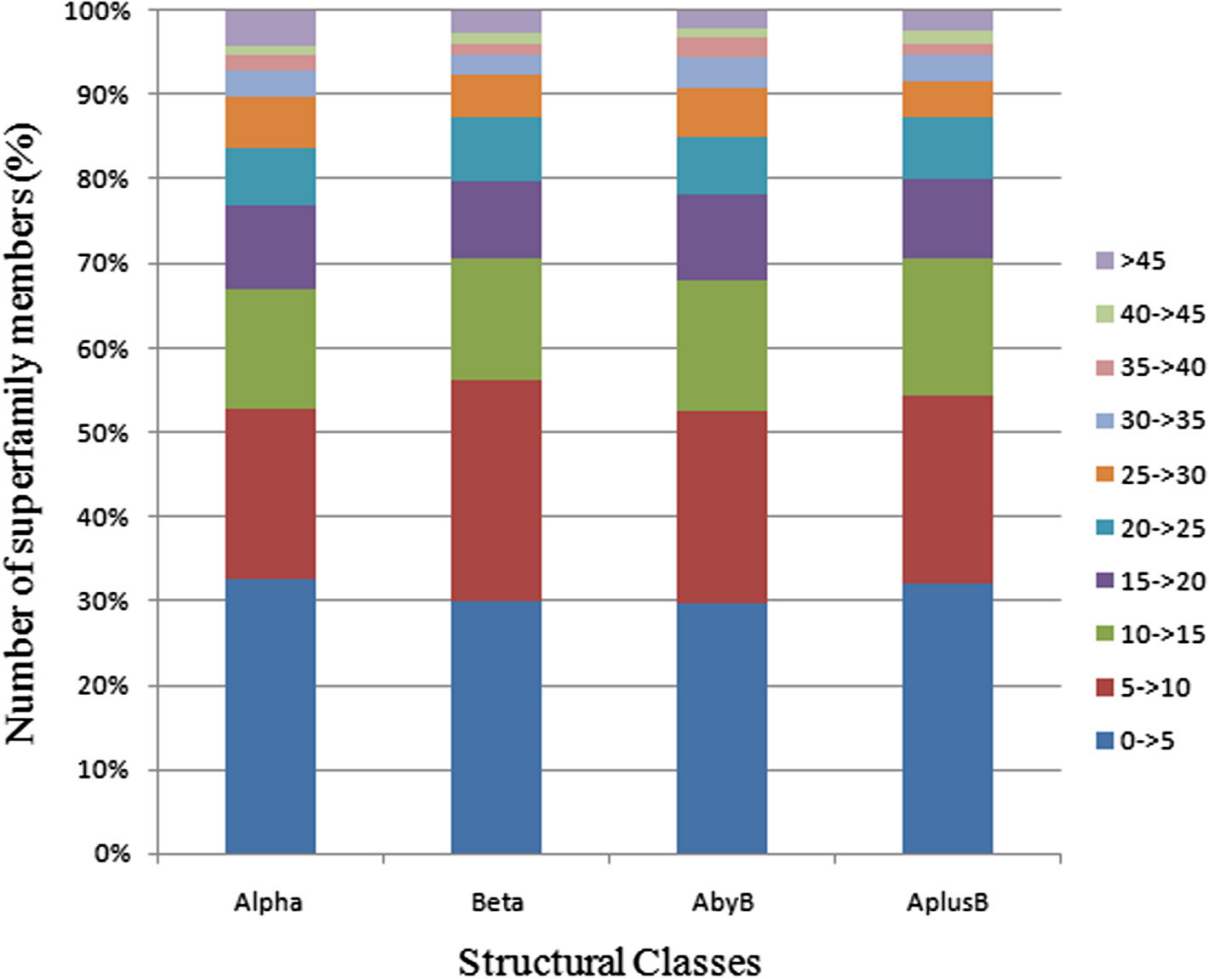
**PASS2 _2008: Class-specific distribution for all superfamilies present in four structural classes.** [Please refer Figure 2b of reference 7 for a similar figure for PASS2_2004].

Likewise, as shown in Figure 6 and its corresponding figure for PASS2.2 (Please refer Figure 2b of reference 7); the distribution of length variation across different structural classes remained quite similar. Whereas 30% of members in PASS2.2 dataset remained within 0-5% length variation, this value had crossed 30% of members in all four structural classes in PASS2.3 dataset. The size of the bin representing 5-10% length variation had increased in case of β class between the two datasets [range = 30-48% for PASS2.2 and 30 -56% for PASS2.3 versions], and bin representing 10- > 15% had reduced marginally in all classes. Bin representing 35- > 40% had reduced in α and β classes, but remained nearly constant in other two classes, >45% length variation bin had also decreased across four SCOP structural classes.

Upon applying previous classification rules (as described in earlier studies [7]) on PASS2.2 dataset, severe reduction of length-deviant superfamilies was noticed (from 64 in PASS2.2 to four in PASS2.3 dataset). Reasons for this severe reduction can be hypothesized as:

a) Cut-off of 75% [% of members in a superfamily should be deviant for the superfamily to be length-deviant] may have been very stringent

b) Newer members have been added and older members have been removed

c) Split of superfamily in new SCOP (1.73)

d) Better alignment quality of PASS2.3 dataset

To remove the above issues and to reduce the subjectivity involved in the previous classification approach [7], an improved classification scheme has been proposed. [As detailed below] Splitting of some superfamilies was noted (Results and Discussion, Section Mapping of superfamilies between two versions of PASS2) and the threshold of 30, 50 and 75% were tried (data not shown) in which 75% was found to be the optimum choice. Also, in order to obtain a consensus, majority of the members (75%) in a superfamily should follow similar trends and hence this threshold was chosen for our analysis.

### Overall changes in PASS2.2 And PASS2.3 Databases

It was observed that 19.5% of the members remained constant between the databases and 45.5% new members were included. Interestingly, 35% of the members in PASS2.2 were excluded in PASS2.3 version [Figure 1B]. The exclusion of members may be a result of being superseded by better resolution crystal structures (of same proteins) or being annotated as outliers (members which could not structurally aligned with the other superfamilies) [11] or removed by PASS2.3 methodology’s stringent entry-level threshold of having less than 40% sequence identity between superfamily members. In order to get a better estimation about the changes occurring in between the two versions, 64 length-deviant superfamilies (derived from PASS2.2 dataset), were taken for objective comparisons and analysed with respect to PASS2.3.

It was observed that at superfamily level, 47 length-deviant superfamilies had acquired more than 60% newly added members, and rest had <50% newly added members. It was also noted that most superfamilies in the new PASS2 database retained only a quarter of old members, while remaining were newly added members in the database. This observation raised an interesting question, as to what happened to the rest of the members (about three quarters in each superfamily)?

In the 64 superfamilies 809 domains in total were present in PASS2.2 dataset, but only 333 domains were carried over to PASS2.3 dataset. On further inspection of the position of the remaining 476 domains, it was established that 162 domains had been filtered out as outliers. Since PASS2.3 dataset was derived from SCOP 1.73 version [3], these members were searched in the SCOP 1.73 database, and noticed that all the members belonged to their respective superfamilies. Therefore, by taking 314 domains as queries, BLAST (Basic Local Alignment Search Tool) and HMM (using HMMER 2.0) searches were done against PASS2.3 dataset and observed that 201 members were picked up as hits from the same superfamilies and while the remaining 113 domains were not associated with any superfamily. Further investigation into 113 missing members was performed by adding them into the corresponding superfamilies of PASS2.3 version and then performing a forcible multiple sequence alignment with CLUSTALW and MALIGN. Percent identity matrices were determined by above methods to gauge the sequence identity of the other old members with respect to the new members. If a particular old member had >30% identity with any other superfamily members, it was placed under the same superfamily; hence 80 (63 + 17) members could be accounted for their original superfamilies. However, 33 other members still could not be accounted in the previous dataset and their absence in PASS2.3 dataset needs to be addressed. A note should also be made about few domains, which though not found in PASS2.3 version in their original form, were actually superseded by better resolution structures and associated with the same superfamilies.

The classification methods (More than 75% of members have to be >30% length-deviant for the superfamily to be called as length-deviant) applied in earlier studies when applied on PASS2.3 dataset did not yield encouraging results due to its subjective nature. In most superfamilies,% of members which were length- deviant (i.e. >30% length variation with respect to average domain size) decreased significantly, especially in ribbon-helix-helix superfamily (SCOP code: 47598) where previously all members were length-deviant, but presently only one-third of members are length-deviant. In only 17% (11 out of 64) of all superfamilies, did the% of length-deviant members increase as compared to the old dataset? Such results called for an improvement in the classification scheme, wherein if ¾ of the superfamily showed greater than 30% of length variation then they were catalogued in “length- deviant”, whereas if they showed <10% length variation, they went into “length-rigid” group and rest were collated in “length-normal” group. The major change introduced here was to deal with the sparsely populated superfamilies with a more relaxed approach (as explained in detail below, Section Improved scheme of classification for PASS2 superfamilies).

### Analysis of 10 length-deviant and 10 length-rigid superfamilies

**Figure 7:**
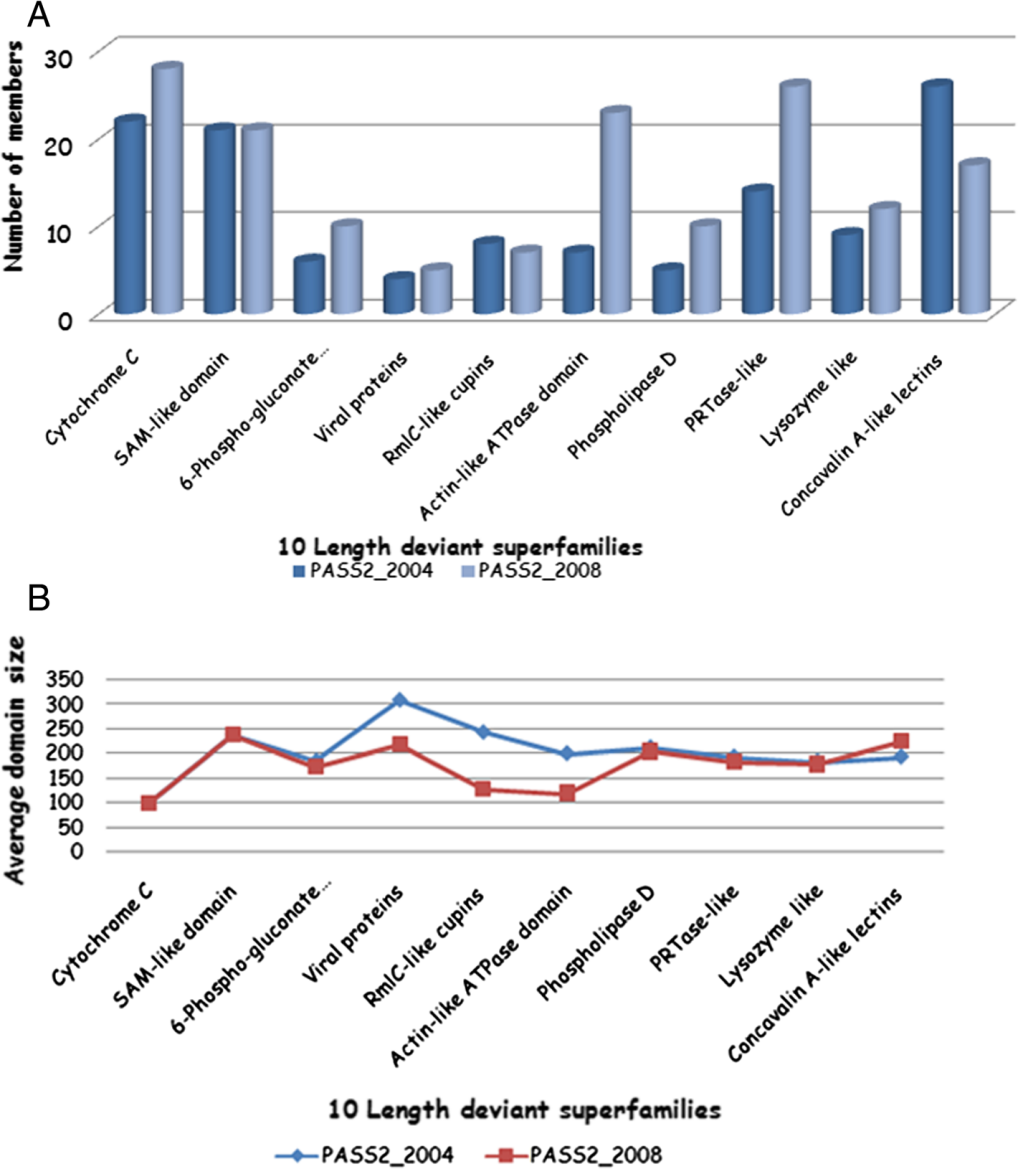
**Length deviant groups tend to accumulate longer members.** These parameters will enable differentiation between the database versions in terms of domain length. **A)** Distribution of number of members in two PASS2 versions for ten length-deviant superfamilies, **B)** Change in average domains size for the ten length-deviant superfamilies.

Discussions about domain member transitions between the databases have been done at coarse-grained level, but to get a closer look, they had to be investigated at fine-grained level, for which the ten length-deviant and length-rigid superfamilies (list obtained from [7]) were selected. The number of members in the ten length-deviant superfamilies had increased substantially (except in superfamilies with SCOP code 49899 and 51182), whereas the average size of the superfamilies seems to have decreased [Figure 7A, B]. Such results showed that these superfamilies may not remain length-deviant with the addition of similar-sized members which had decreased the overall length variation.

A detailed study into the actual members present in each version of superfamilies showed that in length-deviant ones (superfamilies with SCOP code 48179, 49749, 53271 and 53955 etc.), 50% of the total members were new, while other ~50% were retained from PASS2.2 dataset, except in concavalin A-like lectins/glucanases (SCOP code: 49899) and actin-like ATPase domain (SCOP code: 53067) where almost all members were newly added.

Taking up the case of actin-like ATPase domain (SCOP code:53067), this superfamily had seven members, mostly hexokinases in the previous database but presently the number had increased to 23 by addition of other types of kinases as glucokinase, fructokinase, N-acetylglucosamine kinase, pantothenate kinase etc. (which may have been due to the inclusion of newly added structures). New members also included transcriptional regulators (example: MLC protein of ROK family) which controlled the expression of a number of genes encoding enzymes of the phosphotransferase system (PTS) in *E.Coli* [1z6r] [12], exopolyphosphatase/guanosine pentaphosphate phosphohydrolase (PPX/GPPA) enzymes (which played central roles in the bacterial stringent response induced by starvation [PDB code: 1t6c] [13]), inorganic polyphosphate/ATP-glucomannokinase (which used poly(P), a biological high energy compound presumed to be an ancient energy carrier preceding ATP, as a phosphoryl donor and known to function in bacteria [PDB code: 1woq] [14]), Yeaz-like family members (which were essential for bacteria [PDB code: 2a6a] [15]), N-acetylglucosamine kinase (GlcNAc) (which was a major component of complex carbohydrates and was synthesized *de novo* or salvaged from lysosomally degraded glycoconjugates and from nutritional sources [16]) and bacterial rod shape-determining MREB protein from actin/HSP70 family (which assembled into filaments with a subunit repeat similar to that of F-actin (the physiological polymer of eukaryotic actin) [PDB code: 1jce] [17]. Another case study of phospholipase C superfamily (SCOP code 56024) revealed that the number of members had doubled in the newer PASS2, but with the penalty of average domain size reduction by 5%. Newly added members belonged to polyphosphate kinase C-terminal domain family [PDB codes 2o8r, 1xdp] [18] or even phospholipase D [PDB code 1v0w] [19]. While some members (PDB code 1foi) were replaced by better resolution structures (1vow) that retained 100% sequence identity and same sequence length.

### Comparison of 10 length-rigid superfamilies across PASS2.2 And PASS2.3 Datasets

**Figure 8:**
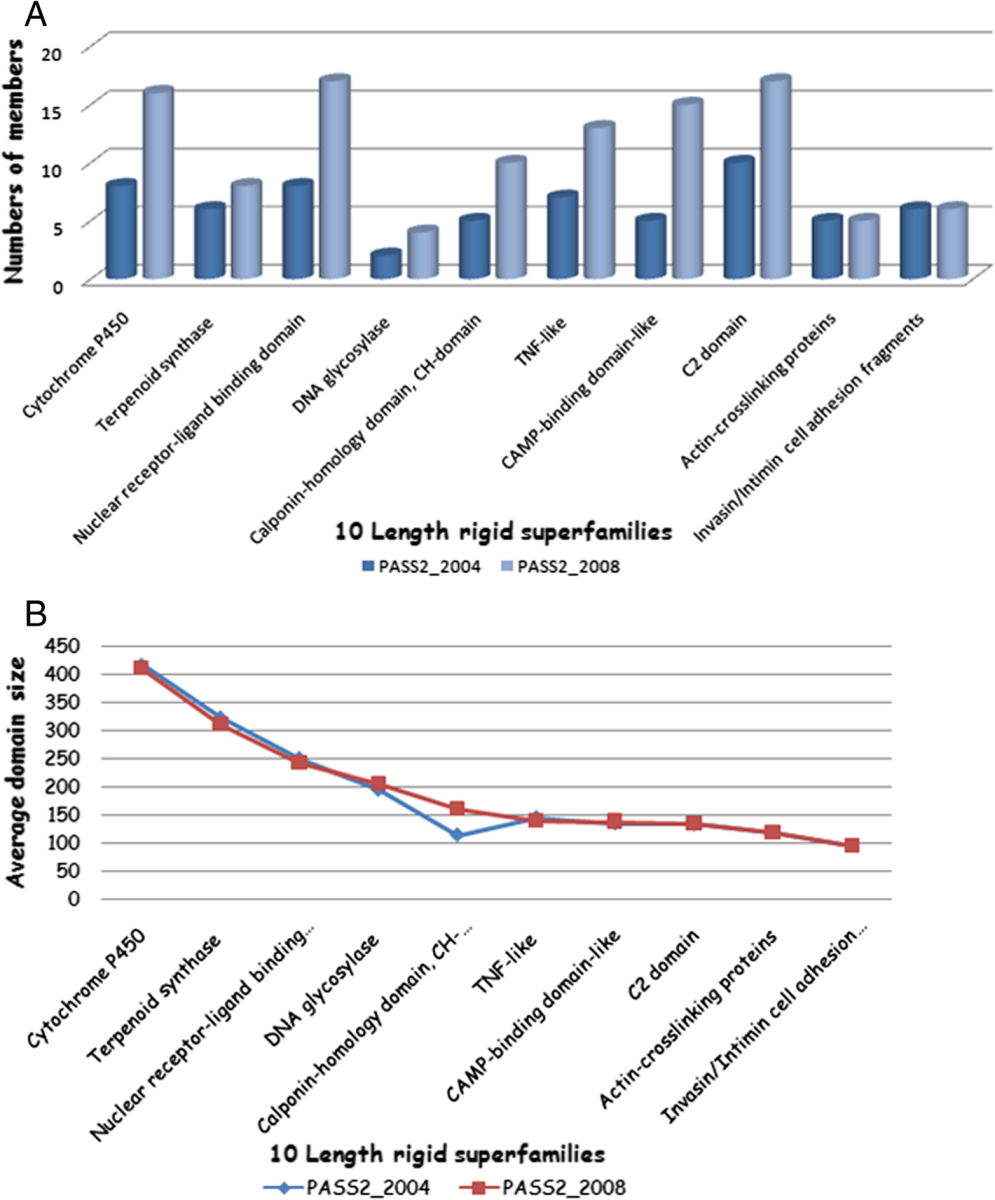
**These parameters will distinguish between the database versions in terms of length-rigid superfamilies. A)** Distribution of number of members in two PASS2 versions for ten length-rigid superfamilies, **B)** Change in average domains size for ten length-rigid superfamilies.

In case of length-rigid superfamilies, though the number of members in each superfamily had risen in the newer dataset, the average domain size consistently remained the same [except for 47576] [Figure 8A, B]. Most superfamilies had no outliers in PASS2.3 version and additions of newer members were seen to be more prevalent than retention of old members (from PASS2.2 dataset), as there was an ~50% increase in the number of members with only 3–4 members being common in both versions. But some superfamilies like actin cross-linking proteins (50405), DNA glycosylase (52141) and invasin/intimin cell adhesion fragments (49373) had almost same members retained in the newer database.

Superfamily like cAMP-binding domain-like [SCOP code: 51206] previously had five members and have accumulated presently 15 new members, out of which three members were common in both versions. Cyclic adenosine monophosphate (cAMP) has been known to be a universal second messenger that, in eukaryotes, was believed to act only on cAMP-dependent protein kinase A (PKA) and cyclic nucleotide-regulated ion channels. In this superfamily, new members were all from cAMP-binding domain family but consisted of different proteins like HCN pacemaker channel (1q3e) [20] or transcriptional regulator (1zyb and 2gau) [21,22]. Two old members, 1hw5 and 1rgs, were replaced by new members as they had better resolution.

### Improved scheme of classification for PASS2 superfamilies

Stringent cut-offs and subjectivity in the previous classification, changes in the composition of superfamilies with their newly added members and length variations of such members were some of the factors which made it necessary for a re-classification of the PASS2.3 superfamilies on the basis of length variations. Previous length-variation analysis performed by our group [7,8] determined standard deviations of length variation parameter in all superfamilies and the average length variation for each superfamily and comparing them. However, due to subjective decisions taken in the previous length variation classification, reproduction of the previous results had become difficult, hence in the PASS2.3 database re-classification, previous subjective decisions were not taken into account.

**Figure 9:**
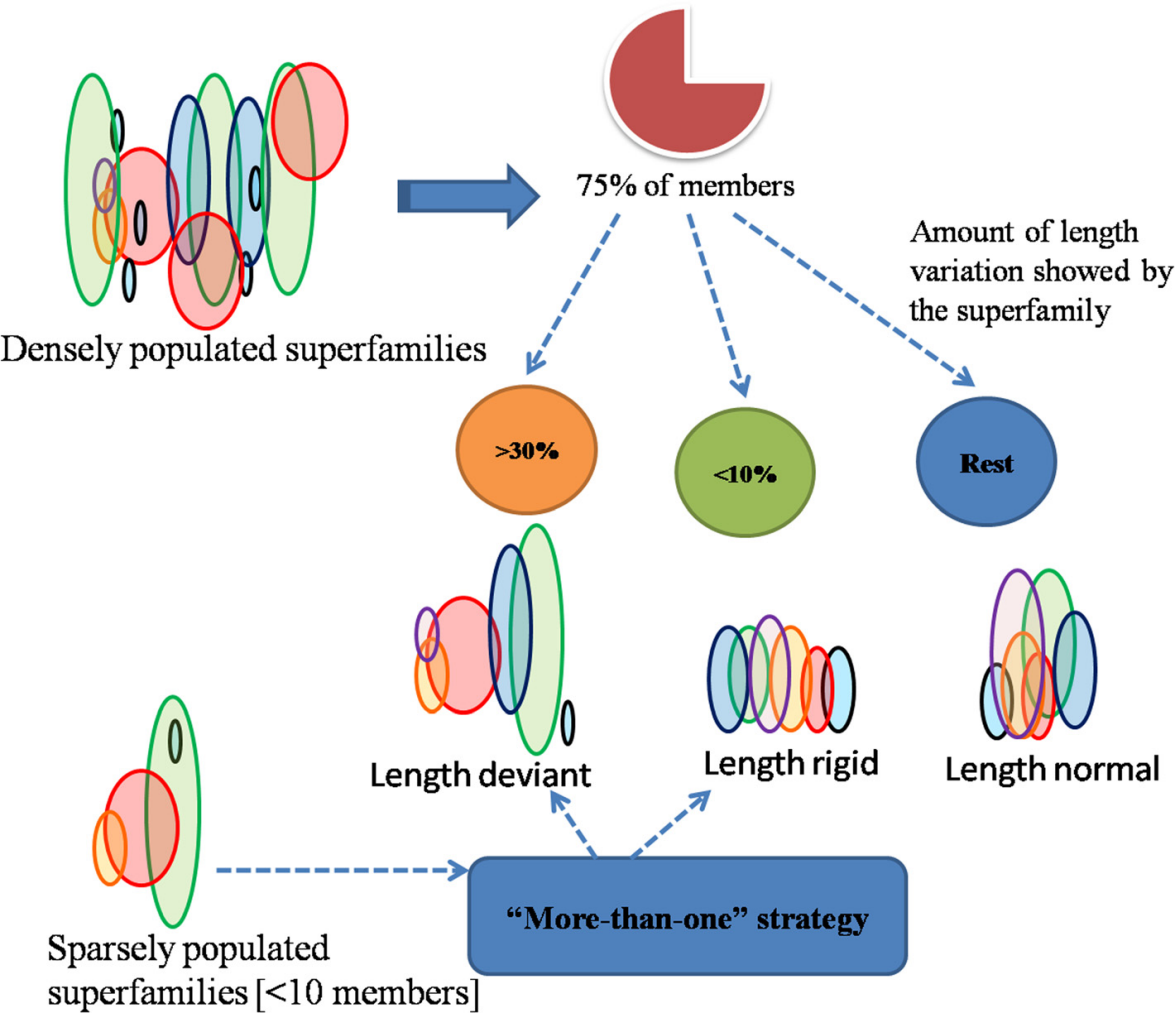
Flowchart of the classification approach used to divide all the multi-membered superfamilies into groups based on their nature of length variations.

Thereafter, an improved scheme of classification of superfamilies was made [flowchart shown in Figure 9]. In this method, the stringent criteria of any superfamily being named as deviant/rigid only if 75% of its members met the rules set, were relaxed and superfamilies with members less than 10 were also given a chance to become length-deviant easily by “more-than-one” strategy [where even one length-deviant member is given the right to make the whole superfamily as length-deviant].

Due to differential motivation in crystallization of proteins, many of the superfamilies were not evenly populated. Since it is not appropriate to apply the same set of rules for classifying highly populated (>10 members) superfamilies along with the lowly populated ones with 75% majority, it was concluded that in highly-populated superfamilies, if 75% of the members showed >30% length variations, then that superfamily was classified as “length-deviant”, while showing <10% length variation led them to be termed as “length-rigid” [Rest members who had 10-30% length variation were included in “length-normal group”].

“More-than-one” strategy: For the less populated superfamilies; number of members showing length variations >30% and <10% were evaluated and whichever group showed majority, that superfamily was allocated to that group. Here, even if more than one member showed length-deviant character, they were termed as “length-deviant” for sparsely populated superfamilies. All the minimal resulting cases of ties were resolved by manual intervention. The present scheme led to length-deviant group, length-rigid group and also led to the formation of “length-normal group”, where superfamilies which neither behaved like deviant nor rigid were grouped into.

According to previously established classification scheme:

PASS2_2004 statistics

Length-deviant: 64 superfamilies

Length-rigid: 24 superfamilies

**Figure 10:**
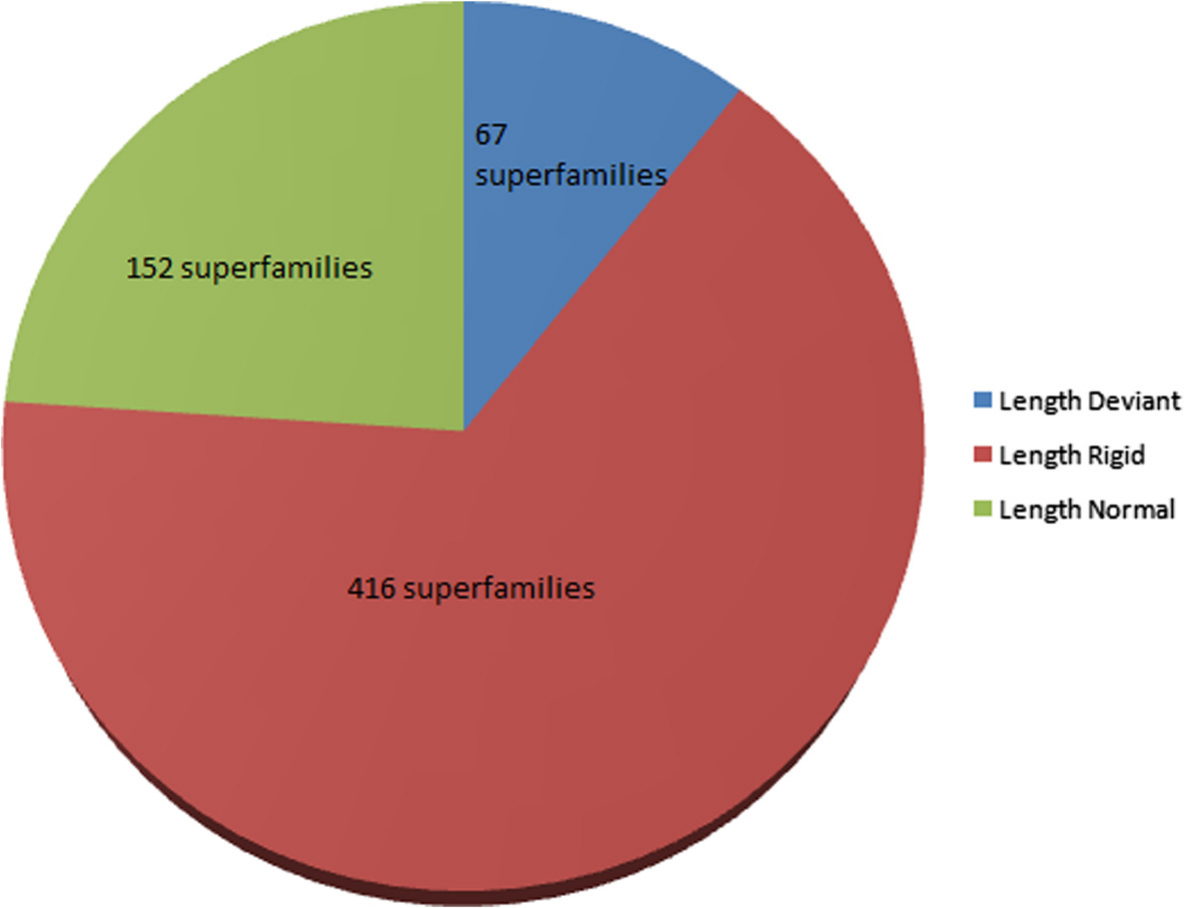
Pie-chart to show the distribution of three groups related to domain length, into which all the PASS2 superfamilies have been distributed after the re-classification.

According to the improvised classification scheme;

**PASS2_2008 statistics: [Figure**10**]**

Length-deviant: 67 superfamilies

Length-rigid: 416 superfamilies

Length-normal: 152 superfamilies [Superfamilies which neither lay in Rigid nor in Deviant groups]

Such an objective classification scheme would help immensely in future for automatic analysis of length variation in cases of updates of databases or even within different secondary databases.

### Case study of length-normal superfamilies in PASS2.2 Dataset becoming length-rigid superfamilies in PASS2.3 Dataset

**Figure 11:**
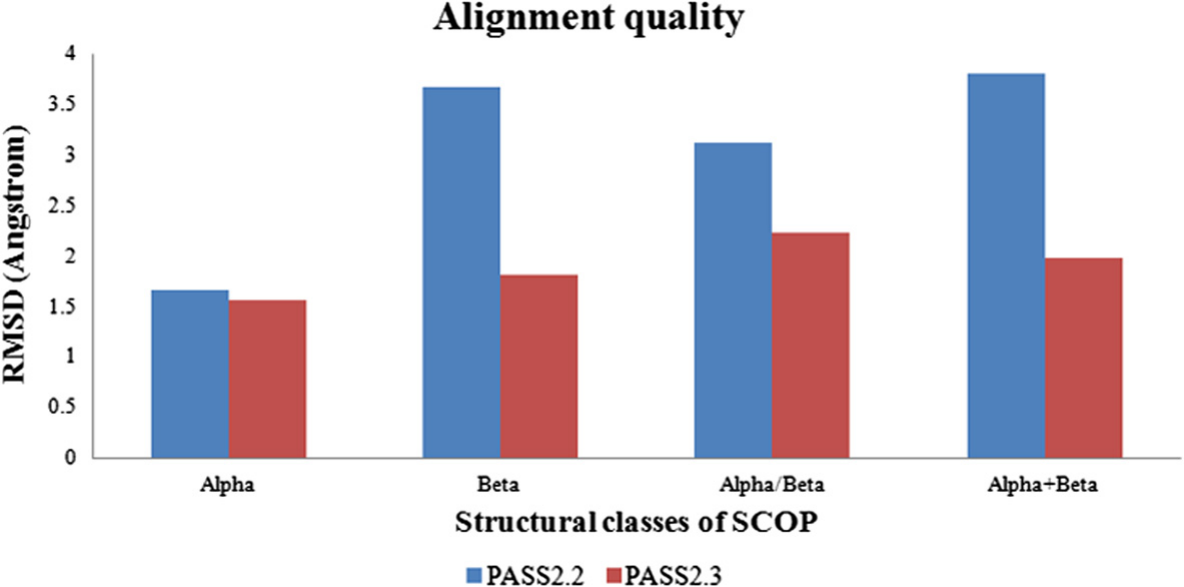
RMSD (root mean square deviation) as an alignment quality indicator used to trace the effect of shift of length-normal superfamilies into length-rigid superfamilies.

It has been observed that several superfamilies have switched their length variant status by changing from length-normal to length-rigid or length-deviant group and vice versa. This may have been a result of different factors. For instance, in order to analyse the alignment quality of the PASS2.3 dataset as one such factor, length-normal superfamilies (which neither belonged to length-deviant nor length-rigid) which had changed groups and became length-rigid were particularly analysed. Approaches such as the number of initial equivalences [Additional file 3: Figure S1], number of secondary structural equivalences (SST) and root mean square deviation (RMSD) of structural entries were used to assess the alignment quality and conservation of length and substructures (Table 1). Higher mean SST equivalence [Additional file 4: Figure S2] in PASS2.3 dataset showed that the amount of secondary structural conservation is similar, while initial equivalence and RMSD [Figure 11] have been major indicators of the quality of alignment which has improved in general in this version (due to improved structural alignment protocols [23]), despite increase in the number of superfamily members. (RMSD parameter was observed to be more contrasting in case of length-deviant superfamilies). It also suggests that the alignments have become more compact, since these superfamilies have “increased in their rigidity” with the addition of members and improvement of alignment. Such refined alignments at the superfamily level can be found rarely and could be further used as reliable evolutionary models to probe various issues of conservation, length variation, structural and functional aspects of protein universe.

**Table 1:** Details of superfamilies taken for case study

**Class**	**Superfamily SCOP code**	**Superfamily name**
α-rich	48225	7-hairpin glycosidases
β-rich	51294	Hedgehog domain
α/β	52743	Subtilisin-like
α + β	54713	EF-Ts domain-like

**Figure 12:**
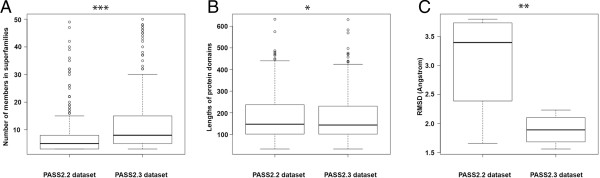
**Summary of changes across different parameters studied at the database level. (A)** Number of members contained in superfamilies [t-test, p-value = 0.001, shown by “***”], **(B)** average domain length of superfamilies [t-test, p-value = 0.056], and **(C)** quality of structural alignments of protein domains in superfamilies [t-test, p-value = 0.093, shown by “**”].

Finally, while the effects of length variation of protein domains are reflected appropriately when studied at each superfamily level, they can be also summarised at full database level [Figure 12A, B, C]. So, while there is significant difference between the number of members contained in superfamilies [t-test, p-value = 0.001, shown by “***” in Figure 12A], domain length did not change to that extent (though in case of certain length-deviant superfamilies, it has reduced) [t-test, p-value = 0.056], and quality of structural alignments of protein domains in the recent version (PASS2.3) has increased significantly [t-test, p-value = 0.093, shown by “**” in Figure 12C] as shown by RMSD calculated between protein domain structures.

## Discussion

The volumes of primary databases such as protein sequence and structural databanks are increasing at a rapid rate. It has been almost impossible to keep track of new, obsolete and similar entries; if not for objective measures such as protein sequence identities. Whereas compendia such as ASTRAL database [24] and earlier PDB_select databases [25] attempt to provide non-redundant entries within an updated version at different levels of sequence identities, it is still non-trivial to compare and appreciate replacement of data and new data that is critical between two updates.

In this study, two versions of PASS2 database updates, i.e. 2004 (PASS2.2) and 2008 (PASS2.3) has been compared, based on many numerical and length variation parameters. Such a detailed follow-up of individual members during two successive updates reveal that many new members have been added and the length of new members have considerably decreased which has led to “increase in rigidity” of the superfamilies. Such studies reflect the length variations found in the structural entries and will be a guiding light in further studies about effect of such length variations upon protein structure and function. Transition dynamics of the domain members were looked in detail at both coarse-grained (superfamily) and fine-grained (member) level, and consequently an objective method for classification of the superfamilies, based on extent of length variation was developed and tested on PASS2.3 (2008 version). Results showed that, *albeit* increasing number of members within a superfamily, the number of length-rigid superfamilies has increased exponentially compared to the length-deviant members, but length-deviant superfamilies remained highly populated in comparison to length-rigid superfamilies. Generally, the number of superfamilies where most members had similar lengths (length rigid) has increased compared with those superfamilies whose members had varying lengths (more than 30% variation from the average domain size of the superfamily). Though this may have been due to crystallization of more similar length (but distantly related as each member has only 40% sequence identity between them) proteins, it can also hint that the superfamilies are becoming more length rigid or having more of average sized members. This may have occurred due to evolutionary reasons, which can be confirmed when their crystal structures are available otherwise by studying their sequence homologues [26].

Each new revision brings about compositional changes in any database hence similar changes were expected to occur in the revised PASS2.3 version also. Further on, the structural alignment methodology varied from PASS2.2 version to PASS2.3 version, which may result in deviation in the results reported earlier by our group. Due to the major changes seen in PASS2.3 version, an objective re-classification of all the superfamilies, with an improved methodology (on the basis of extent of length variation), was performed. This improved scheme will be useful later during automatic analysis of length variation between larger datasets *(Manuscript in preparation)*. An examination of the alignments with various parameters highlighted the increase in quality of alignment in the later versions of PASS2.3 (2008), which will help in linking representative superfamily members to cover rapidly expanding sequence space.

## Conclusion

Each new revision in any database is bound to bring about differences, in the form of newer members, renamed pdb codes, split of superfamilies *etc*. An objective and rigorous study in terms of transition between the database updates and development of an objective classification scheme is required. This analysis will be useful for researchers in designing experiments on specific superfamilies, evaluating the effects of length variations of new members in drug target proteins and in automatic analysis of length variation in cases of updates of databases or even within different secondary databases.

## Abbreviations

PDB: Protein Data Bank; CATH: Class, Architecture, Topology, Homologous superfamily; SCOP: Structural classification of proteins; PASS2: Protein alignments organised as structural superfamilies; CUSP: Conserved Units of structures in proteins; BLAST: Basic local alignment search tool; cAMP: Cyclic adenosine monophosphate; SST: Secondary structural equivalences; RMSD: Root mean square deviation.

## Competing interests

The authors declare that they have no competing interests.

## Authors’ contributions

RS conceived and designed the experiments. ES wrote the necessary scripts and carried out the entire analysis. SSR participated in the analysis. ES wrote the first draft of the manuscript and RS improved it. All authors read and approved the final manuscript.

## Supplementary Material

Additional file 1 Details of Length Deviant (Table S1) and Length rigid (Table S2) superfamilies. Click here for file

Additional file 2 **Details of length variation statistics of length deviant superfamilies between PASS2.2 and PASS2.3 versions.** The table has fields pertaining to SCOP code for superfamilies,Member length, Length variation (%), Average length of members, Members with 30%length variation (%). Click here for file

Additional file 3: Figure S1 Initial Equivalence parameter used to trace the effect of shift of length-normal superfamilies into length-rigid superfamilies. Click here for file

Additional file 4: Figure S2 SST (secondary structural) equivalence parameter used to trace the effect of shifting of length-normal superfamilies into length-rigid superfamilies. Click here for file
