# Supplementary material for: Structural updates of alignment of protein domains and consequences on evolutionary models of domain superfamilies
Source: BioData Min. 2013 Nov 15;6:20. doi: 10.1186/1756-0381-6-20 (PMC4175504; doi:10.1186/1756-0381-6-20)
Supplement: Additional file 1 — Details of Length Deviant (Table S1) and Length rigid (Table S2) superfamilies. [file 1756-0381-6-20-S1.pdf]

SUPPLEMENTARY

Table 1: Details of Length Deviant superfamilies

46626	Cytochrome C
48179	6-Phospho-gluconate dehydrogenase-C terminal domain like
49749	Viral proteins
49899	Concavalin A-like lectins
51182	RmlC-like cupins
53067	Actin-like ATPase domain
53271	PRTase-like
53335	SAM-like domain
53955	Lysozyme like
56024	Phospholipase D

Table 2: Details of Length Rigid superfamilies

47576	Calponin-homology domain, CH-domain
48264	Cytochrome P450
48508	Nuclear receptor-ligand binding domain
48576	Terpenoid synthase
49373	Invasin/Intimin cell adhesion fragments
49562	C2 domain
49842	TNF-like
50405	Actin-crosslinking proteins
51206	cAMP-binding domain-like
52141	DNA glycosylase
