# Supplementary figures and images for: Structural updates of alignment of protein domains and consequences on evolutionary models of domain superfamilies
Source: BioData Min. 2013 Nov 15;6:20. doi: 10.1186/1756-0381-6-20 (PMC4175504; doi:10.1186/1756-0381-6-20)

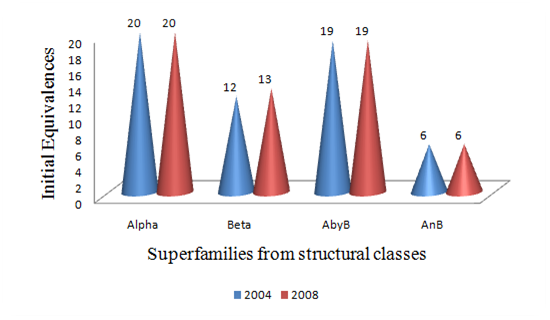

Supplement: Additional file 3: Figure S1 — Initial Equivalence parameter used to trace the effect of shift of length-normal superfamilies into length-rigid superfamilies. [file 1756-0381-6-20-S3.png]

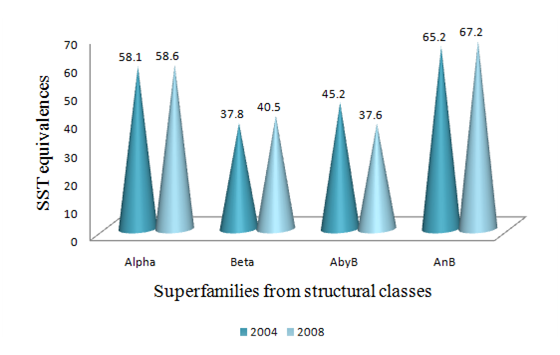

Supplement: Additional file 4: Figure S2 — SST (secondary structural) equivalence parameter used to trace the effect of shifting of length-normal superfamilies into length-rigid superfamilies. [file 1756-0381-6-20-S4.png]
